# The Scope and Impact of the COVID-19 Pandemic on Neuroemergent Patient Transfers, Clinical Care and Patient Outcomes

**DOI:** 10.3389/fsurg.2022.914798

**Published:** 2022-06-09

**Authors:** Josha Woodward, Samuel Meza, Dominick Richards, Lacin Koro, Kevin C. Keegan, Krishna C. Joshi, Lorenzo F. Munoz, Richard W. Byrne, Sayona John

**Affiliations:** ^1^ Department of Neurological Surgery, Rush University Medical Center, Chicago, IL, United States; ^2^ Department of Neurology, Rush University Medical Center, Chicago, IL, United States

**Keywords:** COVID-19, interhospital transfer, neurocritical care, neuroemergency, SARS-CoV-2

## Abstract

**Introduction:**

The SARS-CoV-2 (COVID-19) pandemic continues to substantially alter previously established clinical practice patterns and has transformed patient care in American healthcare. However, studies to evaluate the impact of COVID-19 on neuroemergent patient care and associated clinical outcomes are limited. Herein, we describe the impact of COVID-19 on the Neuroemergency Transfer Program (NTP) - a novel, urban, high volume interhospital patient transfer program.

**Objective:**

To evaluate and describe the clinical impact of the COVID-19 pandemic on the NTP.

**Study Design:**

A single-center retrospective study of prospectively collected consecutive neuroemergent patient transfer data between 2018–2021 was analyzed. Adult patients were divided based upon transfer date into a Pre-COVID (PCOV) or COVID cohort. Patient demographics, transfer characteristics and clinical data and outcomes were analyzed.

**Results:**

3,096 patients were included for analysis. Mean age at transfer in the PCOV and COVID cohorts were 62.4 ± 0.36 and 61.1 ± 0.6 years. A significant decrease in mean transfers per month was observed between cohorts (PCOV = 97.8 vs. COV = 68.2 transfers/month, *p* < 0.01). Total transfer time in the PCOV cohort was 155.1 ± 3.4 min which increased to 169.3 ± 12.8 min in the COVID cohort (*p* = 0.13). Overall mean transfer distance was significantly longer in the PCOV cohort at 22.0 ± 0.4 miles vs. 20.3 ± 0.67 miles in the COV cohort (*p* = 0.03). The relative frequency of transfer diagnoses was unchanged between cohorts. A significant increase in mean inpatient length of stay was noted, 7.9 ± 0.15 days to 9.6 ± 0.33 days in the PCOV vs. COVID cohorts (*p* < 0.01). Ultimately, no difference in the frequency of good vs. poor clinical outcome were noted between the PCOV (79.8% and 19.4%) vs. COV (78.8% and 20.4%) cohorts.

**Conclusion:**

The impact of COVID-19 on current healthcare dynamics are far reaching. Here, we show a significant decrease in interhospital patient transfers and increased length of stay between a Pre-COVID and COVID cohort. Further work to better elucidate the specific interplay of clinical contributors to account for these changes is indicated.

## Introduction

The SARS-CoV-2 (COVID-19) pandemic has substantially altered the traditional means of healthcare delivery in the United States. The direct and indirect effects and implications of the COVID-19 pandemic and related public health crisis brought to the forefront novel challenges in recent healthcare. The impact of COVID-19 on the acute care of patients with time-sensitive neurologic pathologies were likely far reaching. However, to date, a paucity of data to quantify changes in patients, pathologies and clinical outcome for those with neuroemergent diagnoses during the pandemic persists.

Globally, as the number of patients hospitalized with COVID-19 increased, individual and collective healthcare systems were overwhelmed and established a clear need to prioritize and reorganize medical resources and healthcare personal. By April 2020, neurologists around the world were reallocated to care for patients with COVID-19, and neurology beds were reduced to better accommodate escalating volumes of COVID-19 patients (1, 2). As a result, patients with chronic and acute neurological pathology faced increased difficulty to access necessary and critical inpatient and outpatient services (3).

A similar response in the reorganization and reallocation of healthcare resources to escalating volumes of patients with COVID-19 was observed across the United States (4–6). One important and widely documented consequence of the pandemic was a distinct decline in the presentation of neurologic emergencies (5–11). For the first time in recent history, elective hospital admissions and procedures demonstrated a marked decline. Chicago, Illinois was one of the most COVID-impacted regions in the United States. By August 2021, Chicago ranked second and third in COVID-related mortality and overall cases, respectively, with the highest positivity rate occurring between October 2020 and January 2021 (12, 13). Tremendous concern for a catastrophic collapse of healthcare networks was anticipated and a $66,000,000 triage hospital with 2,700 beds was rapidly constructed in the Chicago Exhibition Center (14, 15).

In response to the growing geographic discrepancy between neuroemergent patients and subspecialized providers, the Neuroemergent Transfer Program (NTP) was developed at Rush University Medical Center in Chicago, IL to streamline critical time-sensitive neurologic care. The initial overarching aim of the NTP was the development of a rapid urban interhospital transfer program for patients with acute neurologic emergencies. The NTP continues to achieve a high degree of success and since its inception in 2008 has increased annual transfer volume from 610 to 1,221 patients in 2018 (16). We are to date the single largest transfer center for neurological care in Chicago, IL. As such, we report a significant reduction in total interhospital transfer time as compared to prior published values (17). However, COVID-19 presented new challenges as a novel coronavirus without readily accessible and timely test methods or treatment initially available. These undermined the central premise to reduce interhospital transfer time, as delays in both patient presentation and initiation of the patient transfer process occurred due to the limitations of viral testing. As we have previously reported, these delays in initiation of therapy may translate into progressive neurological decline (18–20). Herein, we aim to evaluate and describe the impact of the COVID-19 pandemic on the NTP – our patients and clinical outcomes.

## Materials and Methods

This is an IRB approved (IRB # 17112802), single academic center, retrospective study of prospectively collected data between January 2018 and February 2021. All consecutive interhospital patient transfers of adults ≥18 years of age and transferred via the NTP were accepted without prior screening of healthcare insurance coverage. For comparative analysis, patients were divided based on the date of transfer into two cohorts - a Pre-COVID (PCOV) and COVID. The PCOV and COVID groups were composed of patient transfers from 1/1/2018 to 12/31/2019 and 4/1/2020 to 2/28/2021, respectively.

April 1, 2020 was selected as the initiation date for enrollment in the COVID cohort as this represented the month when elective surgery at our institution was discontinued and diagnostic laboratory methods for COVID-19 were widely available and used in Chicago, IL. Data from the period between 1/1/2020 to 3/30/2020 was excluded from the study.

A multitude of patient demographics and clinical metrics were captured and include: age, gender, transfer volume, transfer time, transfer distance, transfer diagnosis, length of stay (LOS), and clinical outcome. Categorical determination of transfer diagnosis was based on the primary discharge diagnosis, as this reflected the most informed and accurate diagnosis. Total interhospital transfer time was defined as the interval between initial patient acceptance via phone and arrival at our institution. Total transfer distance was calculated as the shortest ground route to our institution from each individual referral center. Binary stratification of clinical outcome was performed based upon the discharge disposition. A “good” clinical outcome represented patient discharge to a skilled nursing facility, acute rehabilitation or home. Conversely, a “poor” clinical outcome represented patient discharge to a long-term acute care facility, hospice or death from any cause during the index admission.

Statistical analysis of continuous or categorical data was performed with SPSS (*IBM, Version 22*) using either a two-tailed T-test or chi-squared test with *p* < 0.05 establishing significance. Raw data is presented using descriptive statistics, continuous variables as means with ranges and categorical data as frequencies with percentages.

## Results

In total, 3,096 patients (50.4% male, 62.1 ± 0.31 years) were included in the present study. The PCOV and COVID cohorts were composed of 2,347 (50.02%, 62.4 ± 0.36 years) and 749 (51.7%, 61.1 ± 0.60 years), respectively. No significant differences in patient demographics were detected between cohorts (Table 1).

**Table 1:** Pre-COVID and COVID cohort demographic data.

	Sex	Number of patients	Mean age (years)
PCOV	Male	1,174 (50.02%)	60.9
	Female	1,173 (49.98%)	63.9
	Total	2,347	62.4 ± 0.36
COVID	Male	387 (51.7%)	60.5
	Female	362 (48.3%)	61.7
	Total	749	61.1 ± 0.6

In the PCOV cohort, the most common transfer diagnoses included: 1. intracranial hemorrhage (ICH, 21.6%), 2. subdural hematoma (SDH, 13.5%), 3. ischemic stroke (IS, 12.3%), 4. traumatic subarachnoid hemorrhage (tSAH, 11.8%), 5. brain tumor (9.6%). In the COVID cohort, the most common transfer diagnoses included: 1. ICH (20.0%), 2. IS (14.0%), 3. tSAH (11.6%), 4. SDH (11.2%), 5. brain tumor (9.7%) (Table 2). A total of 5 (0.67%) patients in the COVID cohort were received with a primary diagnosis of COVID-19 infection. 2 of these patients expired during the index admission, one of which had a concomitant ischemic stroke. The other 3 patients were labeled with a good outcome.

**Table 2:** Comparison of transfer diagnoses between the pre-COVID and COVID cohorts.

	PCOV	COVID
IS	288 (12.3%)	105 (14.0%)
tSAH	278 (11.8%)	87 (11.6%)
SDH	318 (13.5%)	84 (11.2%)
ICH	506 (21.6%)	150 (20.0%)
EDH	8 (0.3%)	4 (0.5%)
CVT	9 (0.4%)	5 (0.7%)
Brain Tumor	226 (9.6%)	73 (9.7%)
Seizures	180 (7.7%)	52 (6.9%)
Infection	128 (5.5%)	47 (6.4%)
Spine Fracture	38 (1.6%)	12 (1.6%)
Spine Tumor	20 (0.9%)	15 (2.0%)
Other	348 (14.8%)	115 (15.4%)
Total	2,347	749

*IS, ischemic stroke; tSAH, traumatic subarachnoid hemorrhage; SDH, subdural hematoma; ICH, intracranial hemorrhage; EDH, epidural hematoma; CVT, cerebral venous thrombosis*.

**Figure 1:**
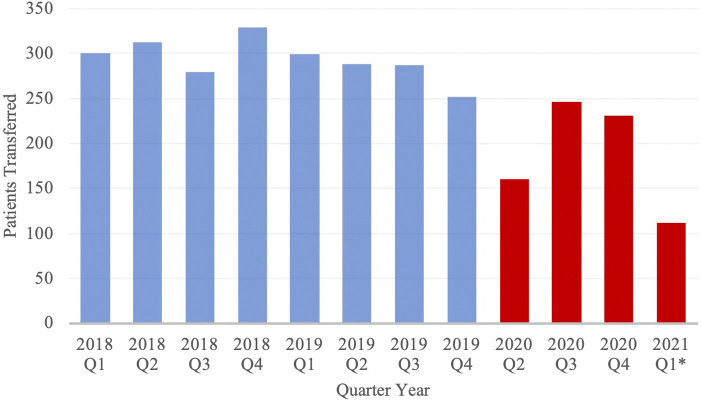
Volume of Neuroemergent Patient Transfers, stratified by Quarter Year. Blue and red columns represent data from the PCOV and COVID cohorts, respectively. *Note: Data from 2020 Q1 has been excluded from the study; Data from 2021 Q1 only includes the months of January and February.

**Figure 2:**
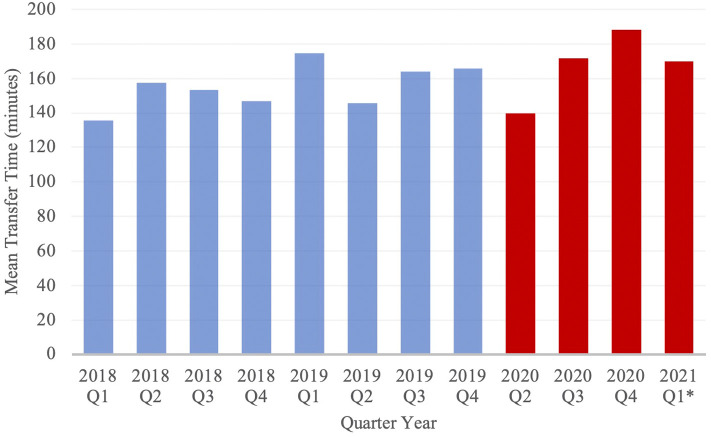
Mean transfer time of Neuroemergent Transfers, stratified by Quarter Year. Blue and red columns represent data from the PCOV and COVID cohorts, respectively. *Note: Data from 2020 Q1 has been excluded from the study; Data from 2021 Q1 only includes the months of January and February.

Transfer volume by month was compared between cohorts (Figure 1). In the PCOV and COVID cohorts, the rates of patient transfers per month were 97.8 ± 2.52 transfers and 68.1 ± 4.46 transfers (*p* < 0.01). Figure 2 depicts transfer data for the two groups, with data divided into discrete 3-month intervals.

Mean total transfer time increased from 155.1 ± 3.42 min in the PCOV cohort to 169.3 ± 12.8 min in the COVID cohort (*p* = 0.21), while mean transfer distance significantly decreased from 22.0 ± 0.4 miles in the PCOV cohort to 20.3 ± 0.67 miles in the COVID cohort (*p* = 0.03). Mean inpatient length of stay during the index admission was significantly increased from 7.9 ± 0.15 days in the PCOV cohort to 9.6 ± 0.33 days in the COVID cohort (*p* < 0.01). In total, 1,873 (79.8%) patients achieved a good outcome in the PCOV cohort as compared to 590 (78.8%) patients in the COVID cohort. Conversely, 455 (19.4%) vs. 153 (20.4%) poor outcomes were observed between the PCOV and COVID cohorts, respectively (Table 3). Taken together, outcomes between the two cohorts were not significantly different (*p* = 0.53). When comparing relative outcomes by month among the COVID cohort, the months of April and March 2020, as well as January 2021, contained the highest percentage of poor outcomes, (ranging from 27.1–31.7%), while August, November and December 2020 contained the lowest percentage of poor outcomes (ranging from 11.3–16.0%) (Table 4).

**Table 3:** Results comparing Mean Transfer Distance, Mean Inpatient Length of Stay, and Outcomes between the two cohorts.^a^

	Mean transfer distance (miles)	Mean travel time (minutes)	Mean inpatient length of stay (days)	Good outcomes	Poor outcomes
Pre-COVID Cohort	22.0 ± 0.4	155.1 ± 3.4	7.9 ± 0.15	1,873 (79.8%)	455 (19.4%)
COVID Cohort	20.3 ± 0.67	169.3 ± 12.8	9.6 ± 0.33	590 (78.8%)	153 (20.4%)
	*p* = 0.03	*p* = 0.13	*p* < 0.001	*p* = 0.53	

^a^ *Note: Patients who absconded or left against medical advice were excluded from Outcomes data in this table.*

**Table 4:** Results comparing Outcomes during the COVID-19 Pandemic by month.^a^

Month	Good outcomes	Poor outcomes
April 2020	67.4%	30.4%
May 2020	72.9%	27.1%
June 2020	83.3%	16.7%
July 2020	79.0%	19.8%
August 2020	88.8%	11.3%
September 2020	78.8%	21.2%
October 2020	77.5%	22.5%
November 2020	87.1%	12.9%
December 2020	80.2%	16.0%
January 2021	66.7%	31.7%
February 2021	75.5%	24.5%

^a^*Note: Patients who absconded or left against medical advice were not counted as either good or poor outcomes*.

## Discussion

The scope and impact of the COVID-19 pandemic on the presentation of patients and pathology, as well as the division and delivery of healthcare resources for patients with acute neurologic emergencies remains poorly characterized. Here, we evaluate and compare two temporal cohorts, a Pre-COVID and COVID group to better understand the implications and impact of the COVID-19 pandemic on the epidemiology of acute neurologic pathology in an urban setting.

No definitive epidemiological census to delineate the beginning of the COVID-19 pandemic in the United States exists. However, the growing incidence of COVID-19 infections coupled with enhanced and widespread public health measures in Illinois prompted an unprecedented cessation of elective surgery at our institution on 4/1/2020. As such, this date was selected as the start of the COVID cohort. Moreover, to provide a washout period to represent a transitional period, data from 1/1/2020 to 3/30/2020 was excluded.

In the present study, no significant difference in baseline patient demographics or characteristics were detected between the PCOV and COVID cohorts. The relative frequency of transfer diagnoses between cohorts were unchanged over the period studied. ICH and spine pathology represented the most and least frequent transfer diagnoses in both cohorts and is consistent with previously reported data (Table 2) (16). The frequency of other common transfer diagnoses, including IS and tSAH, were also congruent with similar studies conducted prior to the COVID-19 pandemic (17, 20–22). COVID-19 infection has been associated with a spectrum of neurologic consequences including but not limited to encephalopathy, acute cerebrovascular disease, and seizure (23–26). Five patients were designated with the primary transfer diagnosis of COVID-19, one of which presented with both COVID-associated pneumonia and an ischemic stroke. This patient expired during the index admission. Nevertheless, neurologic emergencies related to COVID-19 were not a major contributor of patient transfers.

This data demonstrates a significant decrease in the relative number of neuroemergent patient transfers between cohorts and, when stratified by transfer diagnoses, a similar trend in reduced transfer rate. The decreased volume of neurological emergencies is consistent with reports from health systems around the world - both in rural, low-impact and urban, high-impact regions (7–12, 21). There have been several hypothesized reasons to explain this phenomenon, with fear of viral exposure in healthcare settings and adherence to distancing guidelines frequently cited as partial explanations for decreased emergency room visits (27–29). However, the social underpinnings of this observed phenomena are likely complex, multifactorial and beyond the scope of the current discussion.

**Figure 3:**
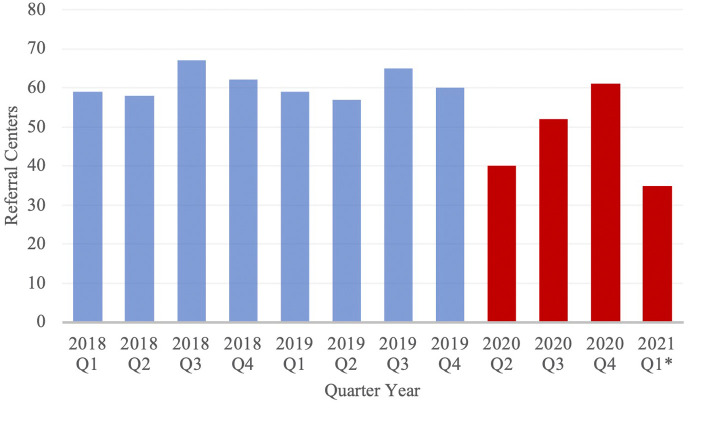
Number of Referral Centers from which neuroemergent patients were transferred, stratified by Quarter Year. Blue and red columns represent data from the PCOV and COVID cohorts, respectively. *Note: Data from 2020 Q1 has been excluded from the study; Data from 2021 Q1 only includes the months of January and February.

The geographic distribution and number of referral institutions were evaluated to better understand alterations in patient referral patterns. The mean total distance traveled was significantly greater in the PCOV cohort. Additionally, during the first quarter of 2020 studied, a notable decrease in referral centers was observed (Figure 3). Taken together, this suggests that during the pandemic, especially early on, COVID-19 had some yet fully characterized impact on patient referral patterns.

Despite the requirement of a rapid COVID-19 test for determination of infection prior to initiation of transfer, no significant increase in total patient transfer time was observed between cohorts. The relatively retained mean total transfer time may, in part, be explained by shorter mean total transfer distance. Furthermore, the time spent obtaining a COVID-19 test may have been balanced by the observed reduction of ground traffic in our urban environment (30). Ultimately, the streamlined transfer process and exclusive partnership with a reputable and reliable regional medical transport company proved successful in maintaining efficiency during the pandemic.

**Figure 4:**
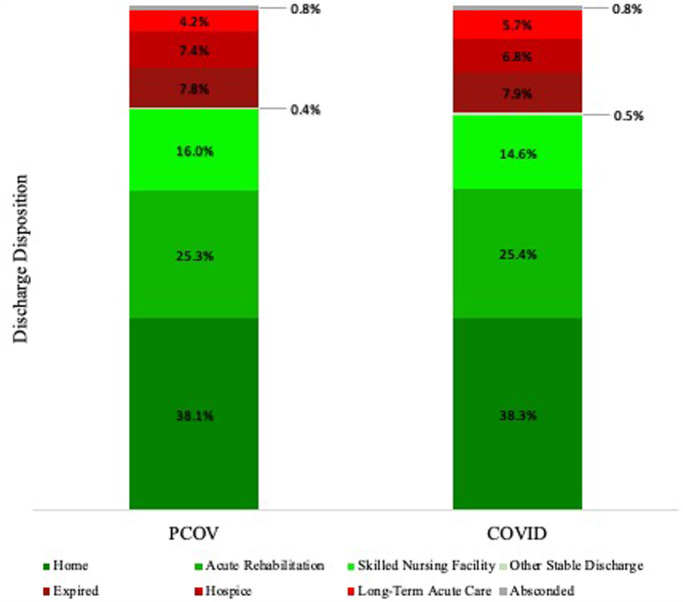
Relative frequency of patient disposition following discharge from our hospital. *Note: Other Stable Discharge includes discharge of medically stable patients to various alternative facilities, including psychiatric hospitals, chemical dependence treatment facilities, and court/law enforcement.

The increase in mean length of stay may result, in part, from patients arriving “sicker” upon presentation. Patients that were hesitant to seek treatment early in the disease course may have waited until their condition got worse before ultimately reaching out to providers. Furthermore, facilities, such as nursing homes and rehabilitation centers, were heavily impacted during the height of the COVID-19 pandemic. Severe staff shortages, growing numbers of infected workers and patients, decreased funding, as well as system-level barriers to receive patients with confirmed COVID-19 infection or symptoms, all contributed to increased stress on post-acute care facilities (31–33). Because these facilities serve an important role in relieving inpatient hospital capacity (34), the increased LOS observed in the COVID cohort may reflect difficulties in discharging patients to such overloaded and constrained facilities during the crisis. However, a difference was not observed when comparing the relative frequency of discharge disposition between cohorts (Figure 4). The reason for the observed increased LOS is likely multifactorial. Further characterization of the clinical contributors that may have led to prolonged hospital stay or differences in patient outcomes is necessary to make definitive conclusions regarding the pandemic’s impact on neuroemergent transfers.

Importantly, based on our binary system of good vs. poor outcomes, no statistically significant difference between our cohorts was noted. In terms of relative outcomes, the worst three months of the pandemic in terms of relative outcomes included April and March 2020 (the initial two months included in the COVID cohort), as well as January 2021. Herein, the relatively high percentage of poor outcomes early in the pandemic correlates with the lowest transfer rates. A similar trend was noted in cases of ischemic stroke in Germany, where hospital admissions were lowest and patient outcomes poorest early in the pandemic before normalizing later as vaccination rate rose and health systems became more experienced with COVID-19 (35). This may support the notion that during this period, patients avoided or delayed seeking care early in their disease course, causing them to present with a more advanced degree of pathology.

Our data is limited in that it does not currently reflect long-term follow-up after discharge. Our binary system of good vs. poor outcomes does not elucidate true outcomes beyond their discharge disposition. Furthermore, our data only reflects patients that managed to enter the health care system. With decreased visits to emergency rooms reported worldwide, little is known about the potential patients who may have suffered from neurologic emergencies and chose not to seek care. In addition, our study only included an analysis of month-wise data during the pandemic, and there is no comparison of such data between cohorts. This serves as a limitation, as both interhospital transfer rates and patient outcomes tend to fluctuate throughout the year independent of the pandemic.

Ultimately, these results demonstrate that even during a pandemic, in one of the most effected geographic regions of the United States, the patients studied did not suffer significant delays in interhospital transfer or worse outcomes. In this, the NTP has been proven efficient and effective in providing outstanding patient care, even during the most turbulent of times in healthcare.

With the emergence of novel COVID-19 variants threatening to lead to surges in cases and prolong the pandemic (36), it would be prudent to remind the public that symptoms of neurologic emergencies should be taken very seriously and to seek care without haste if they arise.

## Conclusion

The impact of the COVID-19 pandemic on the current landscape and dynamics of healthcare are ongoing and far reaching. In this time of unprecedented alteration of healthcare delivery – both with respect to pathology and patients, we show a decreased volume of patients transferred to our hospital, increased length of stay, increased transfer distance and relatively unchanged transfer time and clinical outcomes. However, the specific relationship and/or interplay of contributing clinical factors between the severity of overall illness and constitutional health of patients, increased index hospitalization and clinical outcome remains to be fully characterized. Despite this, the impact of COVID-19 on neuroemergent patients and their clinical care remains unclear. Future work both to reduce the prevalence of COVID-19 and better understand the pathological mechanism and underpinnings of the diverse effects of COVID-19 on the neurological system are warranted.

## Data Availability

The original contributions presented in the study are included in the article/Supplementary Material, further inquiries can be directed to the corresponding author/s.
